# Undifferentiated Pleomorphic Sarcoma of the Breast: A Case Report and Literature Review

**DOI:** 10.7759/cureus.116472

**Published:** 2026-09-18

**Authors:** Mario Della Mura, Filippo Emanuele Angiuli, Erica Ferreri, Vlodymir Munchak, Nicola Porcelli, Alessandro Rizzo, Angela Barile, Giuseppe Broggi, Gabriella Serio, Alda Elena Montanaro, Giuseppe Ingravallo, Gerardo Cazzato

**Affiliations:** 1 Section of Molecular Pathology, Department of Precision and Regenerative Medicine and Ionian Area (DiMePRe-J), University of Bari “Aldo Moro”, Bari, ITA; 2 S.S.D. C.O.r.O. Bed Management Presa in Carico, TDM, IRCCS Istituto Tumori “Giovanni Paolo II”, Bari, ITA; 3 Pathology Unit, A.O.U. Policlinico “G. Rodolico - San Marco”, University of Catania, Catania, ITA; 4 Breast Unit, Policlinico di Bari, Bari, ITA; 5 Section of Pathology, Department of Emergency and Organ Transplantation, University of Bari “Aldo Moro”, Bari, ITA

**Keywords:** breast, rare breast malignancy, sarcoma soft tissue, undifferentiated pleomorphic sarcoma (ups), ups

## Abstract

Primary undifferentiated pleomorphic sarcoma (UPS) of the breast is an extremely rare and highly aggressive soft tissue neoplasm. Representing a diagnosis of exclusion, its rarity and the lack of multicentric studies have prevented the establishment of a clear consensus regarding optimal therapeutic strategies.

We report the case of a 68-year-old woman presenting with a 15-cm bleeding right breast mass. Histopathological examination revealed a proliferation of pleomorphic, atypical cells admixed with numerous osteoclast-like giant cells and a high proliferative index (Ki-67 = 95%). Comprehensive molecular profiling identified pathogenic mutations in *PIK3CA*, *TERT*, *TP53*, and *RB1*, alongside multiple copy number alterations and an overall low tumor mutational burden (TMB: 4.5 mutations/Mb). Due to her unstable clinical condition, the patient did not receive adjuvant therapies and died three months after mastectomy.

A systematic review of the English literature (2002-2026) identified a total of 57 other reported cases of primary breast UPS from 44 different studies. The data underscore the diagnostic challenge of differentiating UPS from more common entities with sarcomatoid features, such as metaplastic carcinoma. Surgery remains the definitive treatment, whereas systemic therapies generally show limited efficacy. Breast UPS is a highly heterogeneous tumor with a poor prognosis.

This report highlights the necessity of integrating clinical, morphological, immunohistochemical, and molecular data to achieve an accurate diagnosis and emphasizes the need for larger case series to uncover tailored therapeutic options for this rare patient cohort.

## Introduction

Undifferentiated pleomorphic sarcoma (UPS), historically grouped under the term malignant fibrous histiocytoma (MFH), is a highly aggressive soft-tissue neoplasm defined by a patternless proliferation of pleomorphic tumor cells with no identifiable line of differentiation [1]. Initial epidemiological observations placed this tumor among the most frequently diagnosed soft-tissue sarcomas in adults; however, this view has been substantially revised. The integration of modern diagnostic methods - such as immunohistochemistry, cytogenetic analysis, and molecular characterization - has revealed that a significant proportion of tumors previously classified as MFH correspond to more precisely defined sarcoma entities [2]. Consequently, UPS is currently considered a diagnosis of exclusion, reserved for cases in which alternative mesenchymal or non-mesenchymal malignancies with dedifferentiated features have been systematically excluded [3,4]. Clinically, this tumor shows no specific site predilection, although it most commonly arises in the deep soft tissues of the limbs and retroperitoneum [3].

UPS can also occasionally involve the breast, posing significant diagnostic and therapeutic challenges. Indeed, numerous breast malignancies can exhibit high-grade sarcomatous features, including more common entities arising from the breast epithelium or specialized stroma, such as metaplastic breast carcinoma and malignant phyllodes tumor (PT). Therefore, an accurate pathological workup, including extensive sampling and careful histological and immunohistochemical evaluation, is required before a diagnosis of breast sarcoma can be rendered [4,5]. Similarly, particularly in patients with a history of breast radiotherapy, other types of sarcoma should be excluded, as a wide range of soft-tissue sarcomas can develop in this setting, most notably angiosarcoma [4,6,7]. Moreover, no well-established recommendations regarding the need for adjuvant therapy exist because of the rarity of this condition and the consequent lack of multicenter studies.

In this report, we present a novel case of UPS of the breast characterized by its very large size and the striking presence of numerous osteoclast-like giant cells, describing the observed morphological, immunohistochemical, and molecular features. Moreover, we provide a comprehensive review of the literature to further investigate the etiopathogenesis, pathological features, and clinicoprognostic correlations of breast UPS.

## Case presentation

A 68-year-old Caucasian woman presented with a large swelling of the right breast for two months. Her medical history was significant for obesity, type 2 diabetes mellitus, dyslipidemia, arterial hypertension, moderate obstructive sleep apnea syndrome, and ischemic heart disease with chronic heart failure. She underwent fine-needle biopsy at another institution for a right breast mass, which was diagnosed as a malignant mesenchymal neoplasm, NOS; no further treatments were administered. Three months later, she was referred to our hospital because the mass had progressively enlarged, presenting as a large, bleeding lesion; thus, mastectomy (without sentinel lymph node biopsy or lymphadenectomy) was performed.

Gross examination revealed a 15-cm tumor located in the central breast, extending from the superficial to the deep parenchyma and infiltrating the superficial layers of the pectoralis major muscle. Histologically, the tumor was composed of pleomorphic, bizarre, atypical cells with markedly irregular nuclei and eosinophilic cytoplasm, almost completely replacing the normal breast parenchyma. Numerous multinucleated osteoclast-like giant cells were present throughout the lesion. The neoplastic cells were arranged in a vaguely storiform pattern within a scant collagenous stroma. Mitotic activity was brisk (up to 10 mitoses/mm² in hotspot areas), including atypical mitotic figures. Extensive necrosis was also observed. No evidence of lymphovascular or perineural invasion was identified. Immunohistochemical analysis did not demonstrate specific lineage differentiation. In fact, tumor cells were negative for CK AE1/AE3, CK MNF116, CK7, CK19, CK5/6, EMA, p63, ER, PR, GATA3, BCL2, CD117, SMA, desmin, caldesmon, CD31, ERG, FLI1, myogenin, MyoD1, MDM2, CDK4, Melan-A, S100, SOX10, CD45/LCA, and CD30. Vimentin showed diffuse positivity. The Ki-67 proliferation index was approximately 95%. Based on the histological and immunohistochemical findings, a diagnosis of primary UPS of the breast was established. Figure 1 shows the histological and immunohistochemical features of the neoplasm.

**Figure 1:**
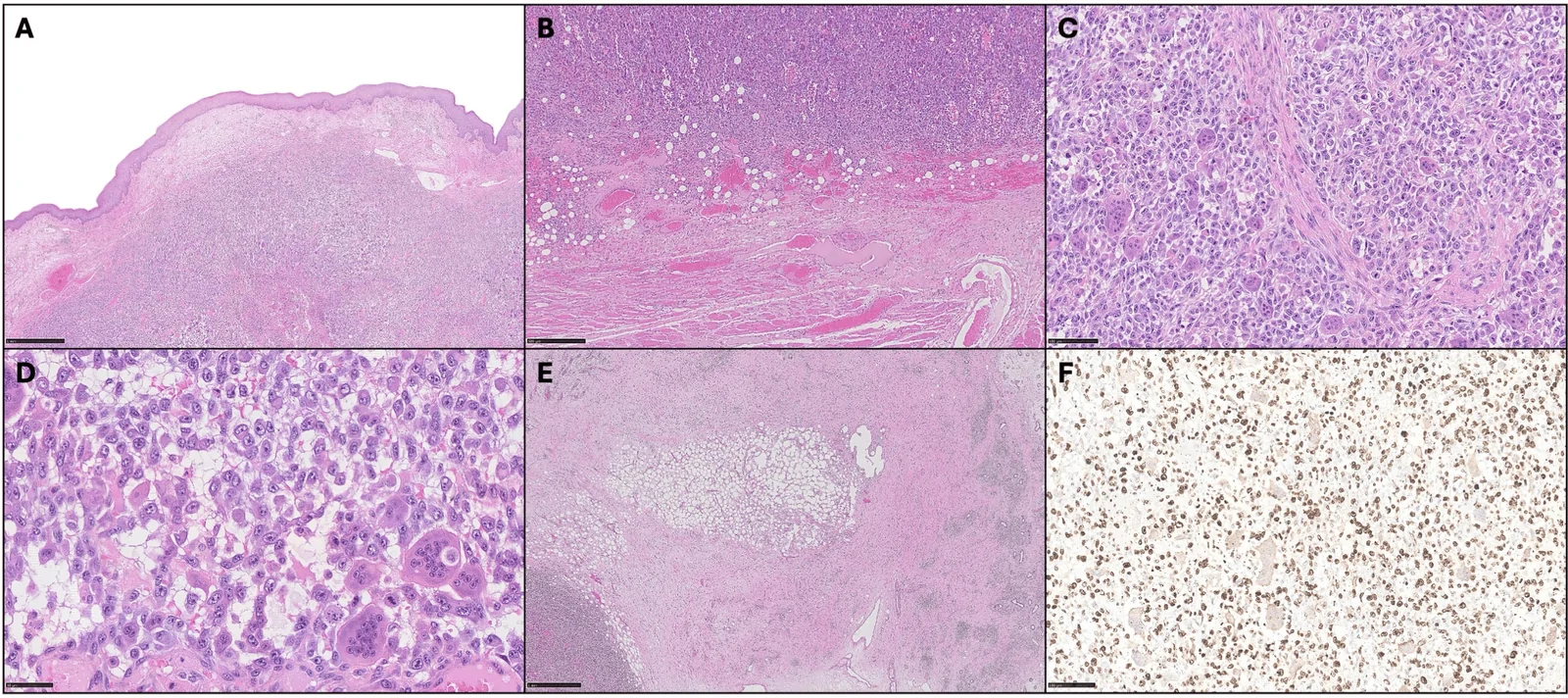
Histopathological features At low-power magnification, a highly cellular neoplasm is seen involving the entire breast and extending from the dermis (A; H&E; scale bar, 1 mm) to the pectoralis major muscle (B; H&E; scale bar, 500 μm). At higher magnification (C, D; H&E; scale bars, 100 μm and 50 μm, respectively), the tumor is composed of pleomorphic, atypical cells arranged in a patternless distribution and embedded within scant stroma, almost entirely replacing the normal breast tissue. Numerous reactive multinucleated osteoclast-like giant cells and mitotic figures are also present. Only scant residual normal breast parenchyma is observed at the margins of the lesion (E; H&E; scale bar, 1 mm). Immunohistochemical staining for Ki-67 (F; IHC; scale bar, 100 μm) demonstrates a high proliferative index of 95%, consistent with the aggressive nature of the lesion.

RNA-based next-generation sequencing (NGS), conducted using the Archer FusionPlex OPBG Custom V2 panel (ArcherDX, Inc., Boulder, CO, USA) to identify gene fusions and hotspot mutations, was negative. Simultaneously, the Illumina TruSight Oncology 500 panel ( Illumina, Inc., San Diego, CA, USA) for hotspot mutations, copy number alterations (CNAs), microsatellite instability (MSI), and TMB detected pathogenic mutations in *PIK3CA* p.(His1047Arg), *TERT* c.-146C>T, *TP53* p.(Cys229Tyrfs10), and *RB1* p.(Arg455). Variants of uncertain significance (VUS) were identified in *POLE* p.(Ile87Val) and *KMT2D* p.(Arg5225Cys). Multiple CNAs were also detected, including copy number gains (four copies) involving *PIK3CA, PRKCI, BCL6, EIF4A2, ETV5, MAP3K13, SOX2, TFRC,* and *TP63*, all mapping to chromosome 3, and single-copy losses affecting *CDK8, DIS3, ERCC5, RB1, CTLA4, CXCR4, KRAS, PIK3C2G, ERBB4, LRP1B, GATA6,* and *PIK3C3*. TMB was 4.5 mutations/Mb. Overall, the molecular findings were consistent with a malignant neoplasm; however, the identified mutations were not pathognomonic of any specific entity.

The patient was planned for chemotherapy and radiotherapy, but she did not receive the treatment due to her unstable clinical condition and died within three months after surgery due to massive hemorrhage.

## Discussion

Primary breast sarcoma is a very rare breast neoplasm, representing about 0.1% of all primary breast malignancies. It arises from the breast mesenchymal connective tissue and includes various histological subtypes; among them, secondary angiosarcomas following radiotherapy are the most common, while primary forms are even less frequent [8-25]. UPS is a type of undifferentiated soft-tissue sarcoma that can occasionally involve the breast, thus presenting as a primary breast sarcoma (10.5%-24% of all primary breast sarcomas [4,26-33]) that is characterized by a nonspecific clinical presentation, exclusion-based histopathological definition, and aggressive clinical course [10,26].

We performed a literature review using PubMed, Scopus, and Web of Science (WoS) databases to identify all cases of breast UPS reported in the literature. The search string was as follows: [breast AND (“undifferentiated pleomorphic sarcoma” OR “malignant fibrous histiocytoma”)] in the title, abstract, and keywords. Only English-language original articles published from 1 January 2002, corresponding to the major revision of the UPS category in the WHO classification of soft tissue and bone tumors, to 1 March 2026 were considered. Other potentially relevant articles were identified by manually checking the references of the included literature. Studies were excluded if they: (i) involved non-breast sites, (ii) did not represent primary breast tumors, (iii) lacked essential clinical or histopathological data, or (iv) described tumors that have since been reclassified as other entities (e.g., myxoid MFH, currently classified as myxofibrosarcoma). Article selection was performed independently by two reviewers (M.D.M. and G.C.) according to the predefined inclusion and exclusion criteria. Any disagreements were resolved through discussion and consensus between the two reviewers. Duplicate records, as well as studies that did not meet the eligibility criteria, were identified and removed manually by the same reviewers. The article selection process for the discussion was conducted in accordance with the PRISMA guidelines and is summarized in Figure 2.

**Figure 2:**
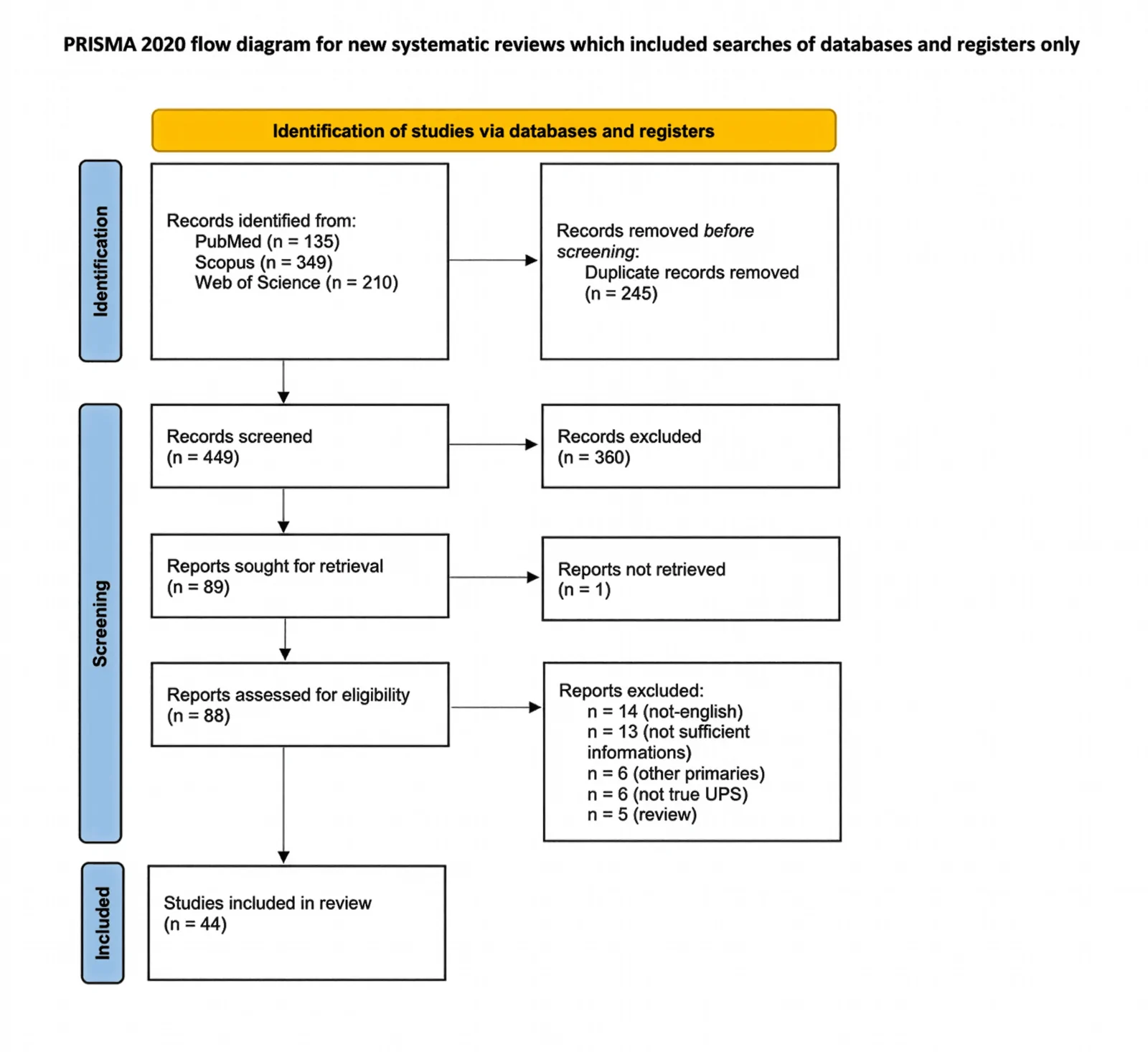
PRISMA scheme of systematic literature review of breast UPS UPS: undifferentiated pleomorphic sarcoma

To date, a total of 57 cases of breast UPS have been reported in the English literature across 44 different articles [4,6,8,10-22,27-29,31,33-56], excluding our case. Table 1 summarizes the most important features of the included studies.

**Table 1:** Table summarizes the most important features of the included studies The table summarizes the 57 cases of primary breast UPS reported in the literature from 2002 to the present, to which we have added the current case. The reports are listed in chronological order and include information on site, the presence of multinucleated osteoclast-like giant cells, history of radiotherapy, treatment, and follow-up. M: male; F: female; R: right; L: left; aCT: adjuvant chemotherapy; aRT: adjuvant radiotherapy; ADF: alive disease-free; AWD: alive with disease; CT: chemotherapy (induction); DOD: died of disease; Ly: axillary lymphadenectomy; mf: multifocal; ICI: immune checkpoint inhibitors; My: mastectomy; nCT: neoadjuvant chemotherapy; n.a.: not avaiable; Re: recurrence; RT: radiotherapy; SLNB: sentinel lymph node biopsy; WE: wide local excission; UPS: undifferentiated pleomorphic sarcoma

Reference	Year of Publication	Age	Sex	Site	History of Radiotherapy	Tumor Size (cm)	Metastasis	Treatment	Follow-Up (Months)	Osteoclast-Like Multinucleated Giant Cell
Yao et al. [37]	2005	72	F	L	n.a.	3.7	NO	My	n.a.	YES
Lesovaya et al. [29]	2025	48	F	L	n.a.	12.5	YES (lung)	My+Ly	6 (DOD)	YES
Wiriosuparto et al. [19]	2003	46	F	R	n.a.	6	NO	My+Ly	8 (ADF)	NO
De Cesare et al. [38]	2005	42	F	L	NO	1.5	NO	My+Ly	288 (ADF)	NO
Quadros et al. [39]	2006	44	F	L	YES	9.5	NO	My-aCT	44 (ADF)	NO
Singh et al. [56]	2007	20	F	L	n.a.	12	NO	My	0.5 (ADF)	NO
Kijima et al. [20]	2007	56	F	R	NO	4	NO	WE+Ly	24 (ADF)	YES
Biswas et al. [40]	2008	46	F	L	YES	3.2	NO	My	n.a.	NO
Balzer et al. [41]	2009	59	F	n.a.	YES	2.1	n.a.	n.a.	16 (ADF)	NO
Choudhury et al. [18]	2010	32	F	R	n.a.	9	NO	My	n.a.	NO
Jeong et al. [42]	2011	76	M	L	NO	3.8	NO	My	12 (Re)	NO
Mahalingam et al. [17]	2011	71	M	L	NO	3.1	n.a.	My	36 (ADF)	NO
Prasad et al. [16]	2011	47	F	R	NO	5	na	My	n.a.	NO
Noh et al. [43]	2012	70	F	L	YES	8	NO	WE	n.a.	NO
Miliaras et al. [15]	2012	73	F	R	n.a.	1.7	NO	WE+Ly	12 (ADF)	NO
Hu et al. [14]	2012	37	F	L	NO	4.5	NO	My+SLNB	72 (ADF)	NO
Balbi et al. [44]	2013	50	F	R	NO	10	NO	My	15 (ADF)	YES
Sang et al. [35]	2021	60	F	L	NO	6.3	YES (cerebellum)	WE	0.7 (DOD)	NO
Qiu et al. [13]	2013	68	F	L	n.a.	5	NO	My	145 (ADF)	n.a.
		58	F	L	n.a.	15	YES (lung)	My + aCT	6 (DOD)	n.a.
		63	F	L	n.a.	5	YES (lymph nodes, liver)	My + aCT	7 (DOD)	n.a.
		24	F	L	n.a.	5	NO	My + aCT	145 (ADF)	n.a.
		52	F	R	n.a.	13	NO	My + aCT	n.a.	n.a.
		20	F	L	n.a.	3	NO	My	n.a.	n.a.
		73	F	L	n.a.	4	YES (lymph nodes, lung)	My	26 (DOD)	n.a.
		51	F	L	n.a.	4	YES (lung)	My + aCT	8 (DOD)	n.a.
		48	F	L	n.a.	17	NO	My + aCT	17 (DOD)	n.a.
Kocak Uzel et al. [45]	2013	37	M	L	NO	12	NO	WE + aRT	42 (ADF)	NO
Liu et al. [12]	2013	48	F	R	NO	7	YES (multiorgan)	WE + aCT	2 (DOD)	NO
Tashjian et al. [10]	2013	72	F	L	NO	3	YES (lung)	My	2 (AWD)	NO
Suhani et al. [21]	2014	45	F	R	NO	12	NO	My + Ly + aRT + aCT	6 (ADF)	NO
Nwashilli et al. [46]	2014	13	F	R	n.a.	6	NO	WE + aCT	48 (ADF)	NO
Harsh et al. [11]	2015	50	M	R	n.a.	3	NO	My	36 (ADF)	YES
Damle et al. [47]	2016	70	M	L	NO	10	NO	WE	6 (ADF)	NO
Srinivasamurthy et al. [48]	2016	29	F	L	n.a.	7	n.a.	My	n.a.	YES
Yamazaki et al. [22]	2018	55	F	R	NO	5	YES (lung, bone)	nCT (uneffective) + M	4 (DOD)	NO
Hatta et al. [27]	2019	38	F	R	YES	18	NO	nCT + M	48 (ADF)	NO
Komaei et al. [49]	2019	63	F	L	YES	n.a.	n.a.	WE	n.a.	n.a.
Kong et al. [4]	2020	75	F	R	YES	5.6	NO	WE + aRT	15 (ADF)	NO
Oh et al. [36]	2004	51	F	L	n.a.	9	YES (lung, brain)	nCT + M + aCT	8 (AWD)	NO
Alhajri et al. [50]	2022	55	M	R	n.a.	11.9	NO	My+aCT+aRT	2 (Re)	NO
Gambichler et al. [8]	2023	58	F	L	n.a.	n.a.	YES (lymph nodes, lung)	My + ICI	15 (DOD) (patient died from disease)	NO
Yam [51]	2022	53	F	R	n.a.	18	NO	nCT + M + aCT	12 (ADF)	NO
Zhang et al. [52]	2023	66	F	R	n.a.	3	n.a.	WE	n.a.	NO
Anzali et al. [33]	2023	56	F	R	n.a.	n.a.	NO	nCT + M + Ly	1 (DOD)	NO
Chakrabarti et al. [34]	2013	58	F	L	n.a.	n.a.	YES (lung)	CT + RT	0 (DOD)	n.a.
Chandrasiri et al. [28]	2024	75	F	R	NO	2.1 (mf)	n.a.	My	43 (ADF)	NO
Žatecký et al. [31]	2023	57	F	L	YES	2.4	NO	WE	68 (Re)	NO
		50	F	L	YES	5	NO	WE	60 (ADF)	NO
		32	F	L	YES	3	YES (lymph nodes)	My	7 (AWD)	NO
Jafarian et al. [53]	2024	50	F	R	n.a.	4.5	NO	My + SLNB + aCT	2 (ADF)	YES
Alshehri et al. [6]	2025	31	F	L	YES	n.a.	NO	WE + aCT	18 (ADF)	n.a.
		36	F	L	YES	n.a.	YES (lung)	CT	9 (AWD)	n.a.
		60	F	L	YES	n.a.	NO	WE	12 (Re)	n.a.
		66	F	L	YES	n.a.	NO	WE	84 (DOR)	n.a.
Liu et al. [54]	2025	37	F	L	n.a.	5.6	NO	My + aCT	3 (ADF)	n.a.
Bahida et al. [55]	2025	56	F	R	YES	4.7	NO	WE	12 (ADF)	NO
Present Case	2026	68	F	R	NO	15	NO	My	7 (DOD)	YES

Affected patients are more frequently female (52 females vs. 6 males), with a mean age of 52 years (range: 13-76). A predilection for the left breast is noted (36 vs. 21).

Clinically, they usually present as rapidly growing masses, typically within a few months, frequently ulcerated, without distinctive macroscopic features, ranging in size from 1.5 to 18 cm, with an average of 7 cm. Occasionally, the presence of neoplastic fever and systemic symptoms (general malaise, weight loss) has been reported [8]. Imaging techniques, such as mammography, ultrasound, and MRI, are incapable of distinguishing breast sarcoma from other tumors. Mammography usually shows a hyperdense mass that can be either well- or ill-circumscribed, usually without calcifications. On ultrasound, the tumor is usually hypoechoic, heterogeneous in texture, with indistinct margins and internal vascularization. On MRI, tumors are usually hyperintense on T2-weighted images, with irregular margins, inhomogeneous enhancement, and restricted diffusion. Thus, the main role of imaging techniques is to provide information regarding local invasiveness, current lymph node status, and distant metastases [4].

Given the absence of specific clinical and imaging findings, histopathological examination remains the gold standard for definitive diagnosis, which can be established only by identifying a proliferation of markedly atypical pleomorphic cells arranged in storiform, fascicular, or patternless patterns, without evidence of a specific morphological or immunohistochemical line of differentiation [1-56]. The presence of osteoclast-like reactive giant cells, ranging from scant to numerous, is observed in a fraction of UPS cases of the breast, occasionally accompanied by osteoid deposition, defining a morphological variant [10]. In particular, they represent around 14% of cases (8/57), without an evident clinicoprognostic correlation. Immunohistochemically, neoplastic cells usually show positivity for vimentin; variable reactivity for histiocytic markers, such as CD68, alpha-1-antichymotrypsin (ACT), alpha-1-antitrypsin (A1AT), and CD163 [4,10-19]; and negativity for other lineage-specific antigens. Occasionally, focal and/or weak reactivity to smooth muscle markers, predominantly SMA [13,21,22], or melanocytic/neural markers, such as S100 [11,13,14,18,22] or Melan-A [8], can be observed; however, such findings should not be interpreted as true myogenic or neural/melanocytic differentiation, which is further supported by negativity for other lineage-specific markers. The Ki-67 index is variable.

UPS may present considerable diagnostic challenges, as a wide range of mesenchymal and non-mesenchymal neoplasms must be excluded. Given the extreme rarity of primary breast sarcomas, breast tumors with sarcomatoid features more commonly represent metaplastic carcinoma or malignant PT, both of which should be ruled out first [8].

In particular, metaplastic carcinoma is a rare, aggressive neoplasm and represents the most frequently encountered malignant sarcomatoid/spindle cell tumor of the breast. It comprises a malignant epithelial and/or mesenchymal component with variable proportions and may occasionally appear entirely sarcomatous in morphology (monophasic) [23]. In this context, careful morphological evaluation and extensive immunohistochemical analysis are essential, as the presence of foci of invasive breast carcinoma or ductal carcinoma in situ (DCIS), along with positivity for epithelial markers such as CKs, p63, or breast-associated markers like GATA3, supports a diagnosis of metaplastic breast carcinoma [24]. It is important to note that metaplastic breast carcinoma often loses expression of one or more epithelial markers, necessitating the use of a broad panel of CKs. Conversely, some sarcomas may show aberrant CK positivity. In such cases, CK positivity primarily favors metaplastic carcinoma over sarcoma, unless it can be clearly interpreted as aberrant within a well-defined sarcoma subtype (e.g., synovial sarcoma, epithelioid sarcoma) [24]. Therefore, a diagnosis of breast UPS over metaplastic carcinoma can only be made when no carcinomatous component (in situ or invasive) is present and multiple CK stainings are negative, as in our case.

PTs are rare fibroepithelial breast neoplasms with a characteristic leaf-like architecture, accounting for 0.3%-1% of all primary breast tumors [23,25]. PTs are classified as benign, borderline, or malignant based on stromal morphology, the latter being the rarest [23,25]. Due to stromal atypia, cellularity, and mitotic activity, malignant PTs can be mistaken for primary breast sarcomas. In such cases, the differential diagnosis relies on identifying residual epithelial structures, typically forming “leaf-like” projections, and the presence of a lower-grade component [25], both of which are lacking in our case. Moreover, stromal cells in PT usually express specialized breast stromal markers, such as Bcl2, CD117, and ER-beta [26], which were completely negative in our case.

Among genuinely primary sarcomas, the main entities to exclude are fibrosarcoma, angiosarcoma, pleomorphic leiomyosarcoma, pleomorphic rhabdomyosarcoma, pleomorphic liposarcoma, and dedifferentiated liposarcoma [3,20,56].

Fibrosarcoma may occasionally display a storiform architecture, yet it does not show marked cellular pleomorphism or multinucleated giant cells. Pleomorphic leiomyosarcoma and pleomorphic rhabdomyosarcoma can be distinguished from UPS by identifying intensely eosinophilic cytoplasm, longitudinally arranged myofibrils, cross-striations, and diffuse positivity for multiple myogenic immunomarkers (e.g., desmin, myogenin, and MyoD1). Although pleomorphic liposarcoma can feature numerous bizarre neoplastic cells, its defining characteristic is the identification of pleomorphic, multivacuolated lipoblasts, which may express CD34 and S100. Dedifferentiated liposarcoma develops from an associated well-differentiated liposarcoma component, typically detectable at least focally at the tumor periphery, and is characterized by amplification of the MDM2 and/or CDK4 genes.

In rare cases, certain non-mesenchymal malignancies may mimic the morphology of UPS, including melanoma and anaplastic lymphoma. Therefore, performing immunohistochemical testing for at least two melanocytic markers (such as SOX10 and Melan-A), together with hematolymphoid markers such as CD45/LCA or CD30, is useful to rule out melanocytic or lymphoid lineage [3,22].

The etiology of UPS remains poorly understood. Both genetic background and environmental factors may play a role. In the breast, three cases (3/58, 5.2%) were reported in patients with tumor predisposition syndromes (Cowden syndrome, Li-Fraumeni syndrome, and hereditary breast and ovarian cancer syndrome (HBOC)) [6,27]. By contrast, 16 patients (16/58, 27.6%) had a documented history of prior radiation therapy to the breast for invasive carcinoma, thus representing radiation-induced breast sarcomas (RIBSs). RIBSs constitute a rare but challenging category, comprising heterogeneous high-grade mesenchymal tumors, including angiosarcoma (the most common), but also UPS and other sarcomas. Diagnostic criteria require a histologically distinct sarcoma arising within a previously irradiated field after a latency period, typically 5-10 years, although this interval may be shorter in predisposed individuals [6,7]. Finally, a case arising in the setting of prosthesis implantation for cosmetic augmentation has also been reported [28].

Two main, non-mutually exclusive theories have been proposed to explain the histogenesis of UPS. The first suggests that UPS is not a distinct biological entity, but rather a common morphological endpoint shared by different mesenchymal tumors. In this view, various sarcomas may progressively lose differentiation during tumor progression, eventually converging toward a highly undifferentiated phenotype. The second theory proposes that UPS originates from the malignant transformation of primitive mesenchymal stem cells rather than from dedifferentiation of a pre-existing sarcoma. According to this model, the timing of transformation along the differentiation pathway determines the tumor’s phenotype: early events lead to poorly differentiated sarcomas such as UPS, while later events result in well-differentiated forms [29].

The molecular profile of this neoplasm is highly heterogeneous, and no recurrent or defining genetic alteration has been consistently identified. UPS represents one of the most morphologically and genetically diverse subtypes of soft-tissue sarcoma. These tumors display a broad spectrum of genomic alterations, including both gain- and loss-of-function mutations, gene amplifications, chromosomal rearrangements, and copy number changes [29]. Molecular analysis has rarely been carried out in UPS of the breast, with no specific mutations (e.g., *TP53* and *RB1*) consistently reported and a TMB ranging from 1 to 19 mutations per megabase pair [8,30]. In our case, no specific driver mutation characteristic of a defined neoplasm or druggable alteration was identified. Moreover, the TMB was relatively low. These findings preclude the possibility of drawing generalizable conclusions and instead underscore that each case should be evaluated individually. Comprehensive molecular profiling, whenever feasible, is warranted both for diagnostic purposes and for its potential predictive implications.

Because of its extreme rarity, the biological behavior of breast UPS remains poorly understood. Nevertheless, it is regarded as a morphologically high-grade neoplasm associated with an aggressive clinical course. According to the current guidelines for soft-tissue sarcomas, the key point in therapy is complete surgical resection with tumor-free margins. The consideration of adjuvant radiotherapy and systemic therapy should be on an individual basis according to staging and oncologically appropriate margins [31]. In the literature, surgery represents the definitive treatment in the great majority of the cases in our review (55/57, 96.5%). Since lymph node metastases are decidedly uncommon (4/57, 7%), regional lymphadenectomy or sentinel lymph node biopsy does not seem to have an important role in the surgical management of the disease, as metastases are mostly hematogenous (in contrast to breast carcinoma). Radiotherapy is preferentially recommended in the presence of positive or close margins [10]. By contrast, chemotherapeutic agents have not been shown to be particularly efficacious [10]. Although sometimes administered as adjuvant therapy after radical surgery [10], when employed in a neoadjuvant setting, chemotherapy is usually ineffective and has been associated with tumor progression [22,32,33]; therefore, upfront radical surgery is preferable whenever feasible. Gambichler et al. proposed immune checkpoint inhibitor (ICI) therapy in selected cases, as they reported that a high TMB can be a predictor of response to the latter [8]. However, in our case, molecular analysis demonstrated a low TMB, confirming the heterogeneity of UPS also from a molecular perspective and suggesting the need for a tailored therapeutic approach for each case, integrating clinical, radiological, and molecular data.

Follow-up was available in 49 patients in our review, with an average follow-up time of 32 months (range: 0-288 months). Of these, 13 died of disease (13/58, 22.4%), and eight achieved no control of disease or experienced recurrences after initial therapy. Death and/or recurrence mostly occurred within the first two years from diagnosis (mean: 10.5 months; range: 0-68 months). The most common metastatic site was the lung (10/12 metastatic cases), while other locations, such as the central nervous system, liver, or bone, have been only rarely reported [13,22,34,35,40].

## Conclusions

Primary UPS of the breast is an extremely rare neoplasm, and data in the literature remain scarce. Our case underscores the heterogeneity of its molecular features, including a low TMB, despite the aggressive clinical nature of the lesion. Furthermore, the rarity of this entity and the paucity of detailed reports are key factors contributing to the lack of consensus regarding optimal therapeutic strategies. Larger case series are warranted to better characterize the clinicopathological and molecular pathways of primary mammary UPS, ultimately improving the therapeutic landscape for this patient cohort.
